# Wearable Fabric System for Sarcopenia Detection

**DOI:** 10.3390/bios14120622

**Published:** 2024-12-18

**Authors:** Zhenhe Huang, Qiuqian Ou, Dan Li, Yuanyi Feng, Liangling Cai, Yue Hu, Hongwei Chu

**Affiliations:** 1 Department of Geriatric Medicine, Shenzhen Nanshan People’s Hospital, Shenzhen 518052, China; hzh932@126.com (Z.H.); lidan233@mail2.sysu.edu.cn (D.L.); 13602436646@163.com (Y.F.); 2 School of Science, Harbin Institute of Technology (Shenzhen), University Town, Shenzhen 518055, China; 23s058081@stu.hit.edu.cn; 3 School of Chemistry and Chemical Engineering, Harbin Institute of Technology, Harbin 150001, China; 4 Department of Biomedical Engineering, City University of Hong Kong, Hong Kong 999077, China

**Keywords:** wearable sensor, health monitoring, sarcopenia

## Abstract

Sarcopenia has been a serious concern in the context of an increasingly aging global population. Existing detection methods for sarcopenia are severely constrained by cumbersome devices, the necessity for specialized personnel, and controlled experimental environments. In this study, we developed an innovative wearable fabric system based on conductive fabric and flexible sensor array. This fabric system demonstrates remarkable pressure-sensing capabilities, with a high sensitivity of 18.8 kPa^−1^ and extraordinary stability. It also exhibits excellent flexibility for wearable applications. By interacting with different parts of the human body, it facilitates the monitoring of various physiological activities, such as pulse dynamics, finger movements, speaking, and ambulation. Moreover, this fabric system can be seamlessly integrated into sole to track critical indicators of sarcopenia patients, such as walking speed and gait. Clinical evaluations have shown that this fabric system can effectively detect variations in indicators relevant to sarcopenia patients, proving that it offers a straightforward and promising approach for the diagnosis and assessment of sarcopenia.

## 1. Introduction

**Figure 1:**
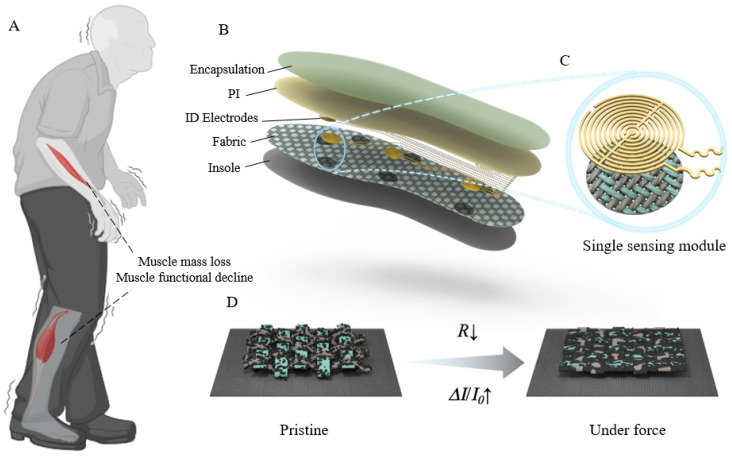
(**A**) Schematic illustration of the sarcopenia patient. (**B**) Exploded-view schematic of the fabric-based piezoresistive sensing system. (**C**) Illustration of a single piezoresistive module that includes a conductive fabric unit and an interdigital electrode. (**D**) Mechanism illustration of the pressure-sensitive response of the fabric-based system.

Sarcopenia was first proposed in 1989 to describe the muscle loss associated with aging [1]. It is characterized by reduced skeletal muscle strength, decreased muscle mass, and impaired muscle function (Figure 1A). Sarcopenia can profoundly impact patients’ daily activities, nutritional intake, and cardiopulmonary functions, and it can also intensify the risk of various chronic disease outbreaks [2,3,4]. Sarcopenia has naturally received growing attention, especially in the context of the aging global population. Accurate detection and effective tracking of the sarcopenia dynamics have been an emergency and pivotal concern.

Current methods for sarcopenia confirmation and assessment are not standardized [5,6,7]; however, clinical practice predominantly relies on direct assessments of muscle strength and mass. Commonly utilized tools include dual-energy X-ray absorptiometry and body composition analyzers. These approaches are often constrained by the requirement for cumbersome instruments [8,9,10]. Some indirect assessment methods can provide a more accessible means for screening and analyzing sarcopenia. For instance, research has indicated that patients with sarcopenia exhibit variations in psychological and behavioral characteristics like walking speed, gait, skin electrical impedance, heart rate, and exhalation, suggesting that monitoring these indicators can effectively reflect the condition of sarcopenia [11,12,13]. Kera et al. conducted a study involving 427 patients with sarcopenia and found that peak expiratory flow rate serves as a reliable predictor of sarcopenia [14]. More importantly, gait speed and other walking tests have been incorporated as one of the clinical diagnostic criteria for sarcopenia in various international consensus guidelines [6]. Nevertheless, these approaches typically depend on rigorous testing protocols, as well as specialized analytical environments and personnel [15,16], making it challenging to achieve long-term, convenient health monitoring and disease tracking for patients.

Wearable electronics provide an effective and promising approach to detect physiological indicators for health-state monitoring in a real-time and in situ manner [17,18,19,20,21]. Some point-of-care mobile devices have been developed and are able to assess some biomarkers of sarcopenia, like motion, skin electrical impedance, and heart rate [22,23]. For example, Leone et al. fabricated a prototype of smart socks by integrating commercial surface electromyography (sEMG) sensors with daily socks for sarcopenia healthcare [24]. These sensors can effectively capture the sEMG signals from the Gastrocnemius–Tibialis muscles of the leg, which can help obtain information about age-related changes in gaits or postures. Kim et al. utilized inertial sensors of both feet to detect the acceleration and angular velocity for better assessment of sarcopenia conditions [16]. Moreover, machine learning models were selected and built to improve the detection performance. As a result, they realized 95% detection accuracy among 10 sarcopenia patients and 10 healthy controls using the sensors and models. These studies demonstrated the promising function of wearable systems for sarcopenia analysis. However, these systems generally feature rigid sensing components, which lead to unstable human–machine interface and uncomfortable application perception [25]. In addition, flexible sensors have also been utilized for sarcopenia screening by monitoring electromyography or walking pressure [23,26]. For instance, Jin et al. developed a wearable multichannel sensor array to detect the surface electromyography for sarcopenia diagnosis [26]. However, complex signal processing and clinical evaluation are still needed for application in real settings. Currently, there is a lack of flexible and portable sensors with sufficient sensitivity and effectiveness for monitoring sarcopenia.

Here, we developed a fabric-based piezoresistive system for motion detection and walking pattern analysis. It comprises a layer consisting of a piece of cotton fabric, an array of interdigital (ID) electrodes lithographically patterned on a polyimide film, and a Kapton encapsulation layer (Figure 1B). The regions of the fabric layer that align with the interdigitated electrodes are coated with carbon nanotube/carbon black (CNT/CB) fillers to create piezoresistive response units (Figure 1C). The pristine fabric possesses an excellent three-dimensional structure that deforms under pressure, resulting in a tighter connection of the conductive fillers within the fabric unit, ultimately reflected in the variations in the electrical signals at the ID electrodes (Figure 1D). Serpentine circuit connections are employed between the ID electrode units, while the Kapton encapsulation layer safeguards against direct contact between the human body and the delicate electrode layer, ensuring stable signal output from the sensor under certain deformation conditions. This fabric-based piezoresistive sensing system can be applied to various parts of the human body for status monitoring, such as voice recognition and heart rate detection. Additionally, it can be directly utilized on the soles of the feet to identify walking speed and gait, providing a feasible and effective approach for real-time and in situ tracking the states of patients with sarcopenia.

## 2. Materials and Methods

### 2.1. Materials

CNT and CB powders were obtained from Shanghai Titan Technology Co., Ltd. (Shanghai, China). Polyvinyl alcohol (PVA) and acetone were purchased from Shanghai Aladdin Biochemical Technology Co., Ltd. (Shanghai, China). Photoresists and the related developers were purchased from the AZ Electronic Materials and Microchem, respectively. Kapton tape was purchased from Shenzhen Chenxi Electronic Technology Co., Ltd. (Shenzhen, China). Commercial fabric substrate was obtained from Huamei Fabric Textile Factory (Foshan, China). Water was deionized before use (18.25 MΩ cm).

### 2.2. Characterization of the Materials and Sensors

The morphology and structure of the fabric were characterized by scanning electron microscopy (SEM) (Zeiss, Jena, Germany, Crossbeam 350). The piezoresistive characteristics were recorded by CHI660e and data acquisition (PL3516/P PowerLab 16/35, AD Instruments, Sydney, Australia, Sampling rate of 10 kHz). The grip intensity was detected by a grip strength tester (Tongfang Health, Beijing, China, CSTF-WL). The body components were determined by body composition analyzer (Tongfang Health, BCA-2A). The humidity modulation was realized in a closed small room, using a dehumidifier (Midea, Foshan, China, CF12BD) and a small humidifier (Xiaomi, Beijing, China, MJJSQ06DY).

### 2.3. Fabrication of the Fabric-Based Piezoresistive Sensing System

The entire system primarily consists of the underlying fabric layer, the intermediate electrode layer, and the upper encapsulation layer. Initially, a small piece of cotton fabric was cleaned three times with abundant acetone and deionized water and then dried at 80 °C for eight hours. A mixture of 100 mg of CNT and 100 mg of CB was mixed into a 1.5 wt% PVA solution and subjected to ultrasonic dissolution. The resulting solution was then drop-coated onto specific locations on the cotton fabric for 7 cycles, and the fabric was subsequently dried under infrared light for six hours. To fabricate the interdigitated electrode layer, Au (200 nm) was first deposited onto a thin PI film (25 μm). Then, the film was spin-coated with a positive photoresist (PR, AZ 5214, AZ Electronic Materials, Luxembourg) at 3000 rpm for 30 s, soft-baked on a hot-plate at 110 °C for 5 min, exposed to ultraviolet light with a specifically patterned mask for 45 s, developed by a developer (AZ 300MIF) for 2 min, rinsed with acetone and DI water, and post-baked on a hot-plate at 110 °C for 5 min. Finally, a commercial Kapton tape was utilized to cover the electrode layer tightly.

### 2.4. Sarcopenia Monitoring Protocols

Participants were recruited from Nanshan Hospital in Shenzhen and surrounding communities. All subjects were classified into sarcopenic patient (SP) and healthy control (HC) groups by professional physicians based on a comprehensive assessment of various indicators. The criteria for diagnosing sarcopenia follow the diagnostic standards proposed by the International Working Group on Sarcopenia (IWGS) [27] and the clinical experience of professional doctors. Detail parameters include grip strength (less than 28 kg for males and less than 18 kg for females), Appendicular Skeletal Muscle Mass Index (ASMI) (Skeletal muscle mass of limbs divided by the square of height, less than 7.0 kg/m^2^ for males and less than 5.7 kg/m^2^ for females), and certain behavioral characteristics. During the experiment, participants placed textile sensors under their feet and followed the physician’s instructions to complete a series of tasks involving walking and standing. Prior to participating in the tests, all subjects were thoroughly informed about the testing procedures and signed an informed-consent form.

### 2.5. Simulation and Statistical Analysis

The finite element analysis simulation was conducted using COMSOL Multiphysics to simulate the actual force conditions of the sensor by mathematical approximation methods [28]. To simplify the demonstration of the plantar pressure distribution during different gait patterns, we assume that in both the toe-out and toe-in gaits, the pressure is uniformly applied to two vertical points on one side of the foot. In normal gait, the pressure is uniformly applied on five peripheral points on the foot. The applied pressure is standardized at 700 N, with displacement used to represent the magnitude of the force. The material science parameters of the fabric substrate are set as 2.1 GPa for Young’s module, 0.4 for Poisson’s ratio, and 1000 kg/m^3^ for density. Statistical analysis of the data was conducted using SPSS Statistical 26.0. T-test was utilized to analyze significant differences in data. A *p* < 0.05 was considered significant.

## 3. Results and Discussion

### 3.1. Characterization of the Piezoresistive Sensor

**Figure 2:**
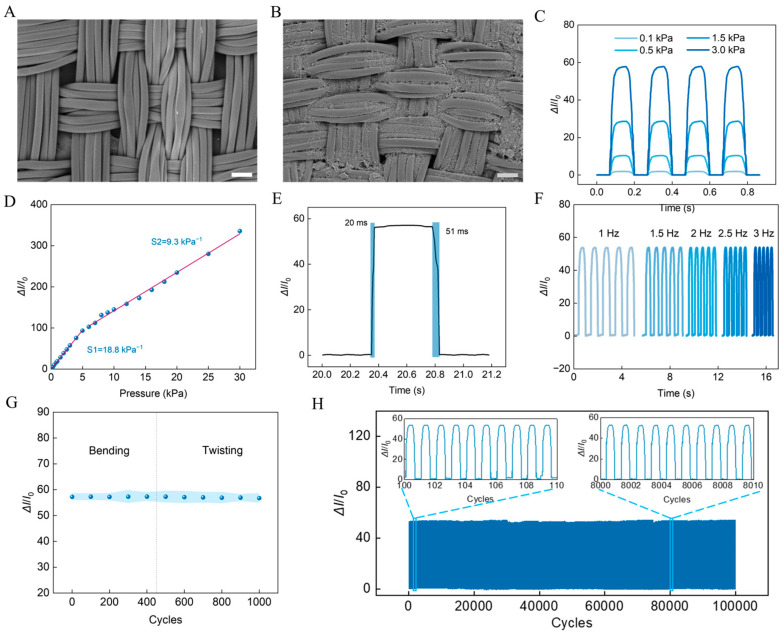
SEM images of the fabric (**A**) without and (**B**) with CNT/CB coating. Scale bar: 50 μm. (**C**) Current signal variations under different applied pressure loads. (**D**) The sensitivity of the fabric-based sensor within the range from 0 to 30 kPa. (**E**) Response and recovery time of the sensor. (**F**) Current signal variations under pressure with different frequency. (**G**) The signal responses of the sensor after bending and twisting processing. (**H**) Long-term recorded signal response of the sensor over 10,000 cycles.

A single piezoresistive sensor that comprises an ID electrode and a conductive fabric unit was utilized to characterize the piezoresistive response toward applied pressure (Figure 1C). Commercial fabric substrates such as cotton or nylon typically possess not only excellent mechanical strength but also feature a three-dimensional interwoven structure and a porous spatial morphology, making them suitable for integration with various fillers to form functional units [29]. We first investigated the influence of drop-coating operation on the signal output. It can be found that the three-dimensional voids of the porous fabric were filled with CNT/CB fillers following the drop-coating process (Figure 2A,B). Furthermore, as the number of drop-coating operations increases, the fillers progressively cover the entire fiber network of the fabric (Supplementary Figure S1). The ID electrode located beneath the fabric consists of several symmetrically arranged fine electrode segments, with minimal spacing between adjacent electrode segments. In the absence of external pressure, the sparse distribution of conductive fillers within the three-dimensional network of the fabric results in a high resistance or complete non-conductivity between the electrode segments. When external pressure is applied to the fabric, the excellent elastic properties of the fabric substrate facilitate a close connection among the conductive fillers within the network, thereby forming a dense conductive layer between adjacent electrode segments. This alteration leads to a sensitive and rapid change in the electrical signal output at the terminals of the ID electrode. The signal response was characterized by the variance in current in the electrode circuit before and after pressure; it was found that the signal output gradually increased with the number of drop-coating applications, reaching a near-saturation level after five cycles of operations (Supplementary Figure S2). The *I*–*V* curves of the sensor under different static pressure loads reveal stable piezoresistive responses to applied pressure (Supplementary Figure S3). It was demonstrated that the current variances increase with the pressure loads (Figure 2C), and the sensitivity of the sensor was determined as 18.8 kPa^−1^ for pressure below 5 kPa and 9.3 kPa^−1^ for pressure from 5 to 30 kPa (Figure 2D). The sensor also exhibited a fast pressure response with a response time of 20 ms and recovery time of 51 ms (Figure 2E), which is mainly attributed to the low tensile module of the fabric and the tight distribution of the conductive fillers in the fabric networks.

Static pressures with different frequencies were applied to the sensor. It can be observed that the sensor can effectively recognize different pressure frequencies (Figure 2F), a capability that is beneficial for the detection of human physiological motions like walking or breath behavior. To characterize the output stability of the fabric sensor, the current variances were recorded after different cycles of bending and twisting. It was demonstrated that the signal of the sensor shows no significant changes over 1000 cycles of deformation (Figure 2G), a feature which arises from the flexibility of the system and the serpentine connection pattern. The influence of environmental humidity has also been considered. We tested the signal response in three distinct humidity environments (30, 50, and 70% relative humidity (RH)). The results indicated that there were no significant changes in the sensor’s output when the humidity conditions were altered (*p* < 0.05) (Supplementary Figure S4). Moreover, continuous pressure operations were carried out with consistent pressure loads. The signal demonstrated excellent stability over 100,000 cycles pressure (Figure 2H). These merits in flexibility and stability favor long-term applications in practical settings.

### 3.2. Performance of the Fabric Sensor for Human Activity Monitoring

**Figure 3:**
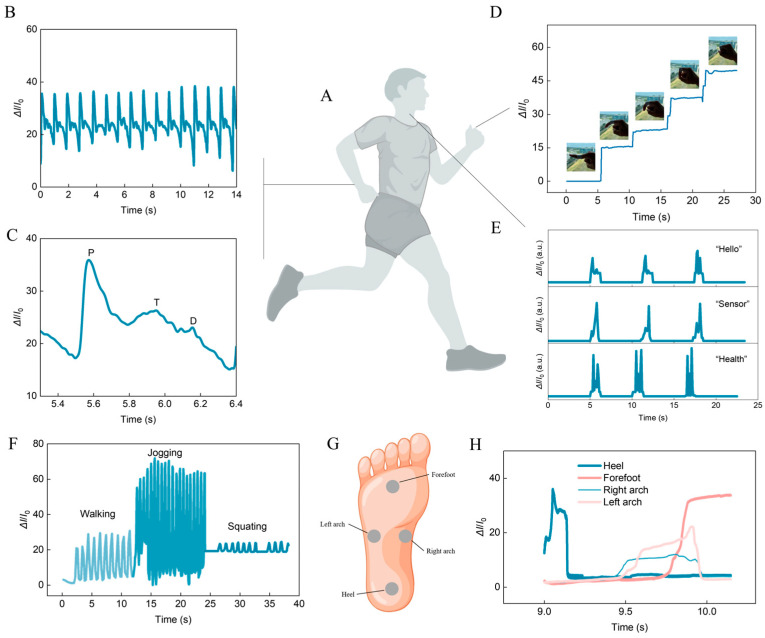
(**A**) Illustration of a person who can be equipped with the fabric-based sensor on different body position for health monitoring. (**B**) The continuously recorded pulse signal. (**C**) Enlarged pulse signal with distinct percussion wave, tidal wave, and diastolic wave characteristics. (**D**) Signal responses for finger bending. (**E**) Real-time current signals respond to different speech actions. (**F**) Signal responses under different exercise conditions. (**G**) Illustration of the work positions on the foot of the fabric-based sensor. (**H**) Real-time recorded signal responses from walking with sensors placed on different parts of the foot.

The excellent flexibility and sensitive pressure detection capabilities of this fabric sensor enable it to be worn on various locations of the human body to record bodily movements and physiological behaviors (Figure 3A). For instance, when the sensor is worn on the wrist, it can successfully achieve continuous readings of pulse signals (Figure 3B). The amplified graph demonstrates that the sensor can sensitively capture three characteristic peaks of the pulse signal, percussion wave (P), tidal wave (T), and diastolic wave (D), and this ability is attributed to the high sensitivity and outstanding conformability of the sensor (Figure 3C). Furthermore, when the sensor is placed on a finger, it was observed that the current variance increases with the degree of finger flexion, and the signal remains relatively stable when the finger is held in that bent position (Figure 3D). During speech, the laryngeal organs and tissues are set into motion, with distinct movement variations corresponding to different words. When the sensor is affixed to the throat, it reveals discernible signal waveforms during the articulation of different words, and there is a notable consistency in the speech signal waveforms for the same word (Figure 3E), indicating that the surface sensor holds potential for voice recognition.

Due to the sensor’s slim profile and lightweight characteristics, it can be positioned between the foot and the insole to detect movement behavior without causing any discomfort to the user. It has been observed that the sensors can effectively identify various physical activities, such as walking, jogging, and squatting (Figure 3F). Furthermore, four sensors were placed at the forefoot, left arch, right arch, and heel, and the signals from all four sensors were recorded during a single step action (Figure 3G). The data revealed that sensors located at different positions on the foot exhibited varying signal strengths and response-curve patterns. When the foot first made contact with the ground, the signal from the heel sensor rapidly increased, reaching its peak quickly, while the signal from the forefoot sensor maximized just before the foot lifted off the ground. The maximum signal amplitude from the sensor placed at arches is slightly lower than that placed at the forefoot and heel, which can be attributed to the differing force distribution across various locations of the foot during walking (Figure 3H) [30,31].

### 3.3. Sarcopenia Monitoring Using the Fabric-Based System

**Figure 4:**
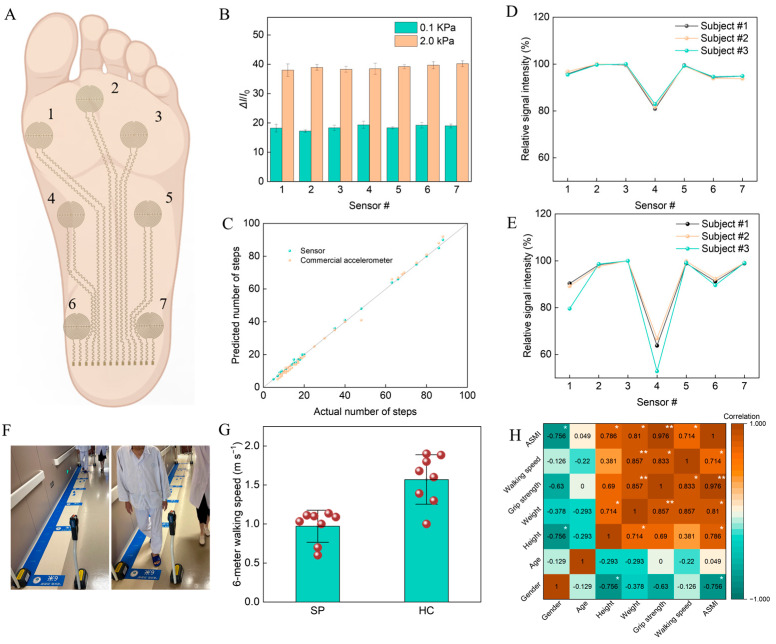
(**A**) Illustration of the sensor array placed on the foot for health monitoring. (**B**) The current variation in different sensor modules under 0.1 and 2.0 kPa loads. (**C**) The correlation between actual step numbers and step numbers predicted by the sensor and commercial accelerometer. Relative signal responses of the sensor array for (**D**) HC and (**E**) SP. (**F**) Images of the 6 m walking-speed test in the hospital. (**G**) Six-meter walking speeds of SP and HC. (**H**) Correlation illustration of various body characteristics. * *p* < 0.05 and ** *p* < 0.01.

To facilitate the diagnosis and assessment of sarcopenia patients, we developed a sensor array integrating seven sensors to map the pressure distribution on the foot (Figure 4A). Initially, we investigated the signal outputs of each sensor when subjected to the same pressure on the integrated platform. The results indicated that there were no significant differences in the responses of the various sensors to the identical pressure load (Figure 4B). We positioned the integrated sensor platform between the foot and the insole, collecting sensor signals during different walking durations. The numbers of steps were calculated by collecting and analyzing the waveform signals from seven separate sensors in a comprehensive manner. The mode of the results from the seven sensors was regarded as the final step number conclusion. This approach could effectively mitigate the impact of any single sensor’s error on the detection results. During the walking trials, we manually calculated the actual number of steps, using a commercial accelerometer for comparison. The findings revealed that the step number calculated by the integrated sensor platform demonstrated a strong correlation with the actual walking data (Figure 4C), exhibiting higher accuracy than the commercial accelerometer in scenarios involving small step numbers.

To analyze the differences in gait between SP and HC, three SP and three HC subjects were recruited, and the sensor signals from their foot while standing were recorded. The relative signal intensities to the maximum signal from seven sensors are illustrated in Figure 4D,E. The results indicated that, for the HC subjects, the pressure intensity detected by all sensors, except for sensor 4, was relatively uniform. In contrast, the signal intensities recorded by sensors 1, 3, and 6 in the SP subjects were significantly lower than those of the HC subjects, suggesting that the foot pressure distribution in sarcopenic patients tends to favor the lateral side. Simulation data were utilized to further elucidate this phenomenon, indicating that individuals with a toe-out gait exert more pressure on the outer side, while those with a toe-in exhibit greater pressure on the inner side (Supplementary Figure S5). This finding suggests that the gait of SP subjects is more inclined toward a toe-out pattern, which is consistent with the experimental observations.

The 6 m walking speed test is a commonly utilized method for diagnosing sarcopenia. In clinical assessments, infrared timers are positioned at both ends of a 6 m pathway, and the walking speed is calculated based on the time difference recorded when the individual crosses the infrared sensors twice (Figure 4F). We conducted this test with eight SP subjects and eight HC subjects, utilizing our integrated sensors to estimate walking speed through the analysis of signal waveforms generated during stepping and stopping. The results revealed a statistically significant difference in walking speed between SP subjects and HC subjects (*p* < 0.05), with the average walking speed of the SP group measuring 1.57 m s^−1^, notably higher than the 0.97 m s^−1^ observed in the HC group (Figure 4G). Furthermore, the walking speed estimated using our integrated sensors demonstrated strong concordance with the results obtained from the infrared timers (Supplementary Figure S6). Additionally, we examined the correlations among various clinical characteristics for the subjects, finding a robust correlation between walking speed and grip strength, as well as ASMI, while no significant correlations were observed with gender, age, height, or weight (Figure 4H). Therefore, employing our integrated sensors for walking speed assessment represents an effective method for monitoring the status of sarcopenia.

As alluded to above, this fabric-based piezoresistive system exhibits promising solutions for sarcopenia assessment through analysis of plantar pressure, walking speed, and gait pattern. The inherent flexibility and lightweight characteristics of the system enable it to function effectively on the human body, providing stable operational capabilities without imposing significant burdens. In comparison to other detection systems based on rigid sensing elements, this system offers a more stable working interface and a more comfortable wearable experience [12,25]. Moreover, due to the unique three-dimensional network structure of the fabric and its favorable elastic modulus, this system demonstrates competitive sensitivity compared to similar piezoresistive sensors, while also offering excellent flexibility and multi-angular analytical capabilities to assist in the assessment of sarcopenia (Supplementary Table S1). Additionally, the fabric system holds the ability to be integrated into everyday garments, such as insoles, thereby eliminating the need for supplementary components often required by other wearable sensors, like patches or rings [2]. The sensor can connect to external analyzers for data measurement via ultra-fine wires, and the use of a flexible processing unit will enhance the overall convenience and practicality of the system, which will be realized in the next phase of device optimization.

## 4. Conclusions

In summary, we developed a flexible wearable sensing system based on conductive fabric and electrode array. This piezoresistive sensor exhibited excellent performance to detect applied pressure with a high sensitivity of 18.8 kPa^−1^ for pressure below 5 kPa. Due to the soft characteristics and great capability for pressure perception, the sensor can be functioned at different body locations to monitor human activities or health-related features, such as pulse rate, finger movement, speaking, or exercise. Moreover, this fabric-based system can be integrated into shoes to detect walking speed and gait for sarcopenia analysis. The results indicated that patients with sarcopenia exhibit a greater tendency toward a toe-out gait, along with a significantly reduced walking speed compared to healthy individuals. Our developed flexible wearable piezoresistive sensing system offers a reliable and straightforward method for real-time, in situ monitoring of SP, meaning that it may have promising value for the clinical diagnosis and treatment evaluation of sarcopenia in the future.

## Data Availability

The original contributions presented in this study are included in the article/supplementary material. Further inquiries can be directed to the corresponding authors.

## Supplementary Materials

The following supporting information can be downloaded at https://www.mdpi.com/article/10.3390/bios14120622/s1, Figure S1: SEM images of the fabric with different coating times; Figure S2: The current variations in the fabric-based sensor with different drop-coating times; Figure S3: *I*–*V* curve under various applied pressure loads; Figure S4: The signal response of the sensor in different humidity conditions. Figure S5: FEA simulation of force distribution with different walking postures; Figure S6: The correlation between walking speed calculated from the sensors and infrared time-meters. Table S1: Performance comparison with other similar works. References [16,23,24,26,29,32,33,34,35,36,37,38] are cited in the supplementary materials.
