# Supplementary material for: Wearable Fabric System for Sarcopenia Detection
Source: Biosensors (Basel). 2024 Dec 18;14(12):622. doi: 10.3390/bios14120622 (PMC11674443; doi:10.3390/bios14120622)
Supplement: Supplementary file 1 [file biosensors-14-00622-s001.zip › biosensors-3330710-supplementary.pdf]

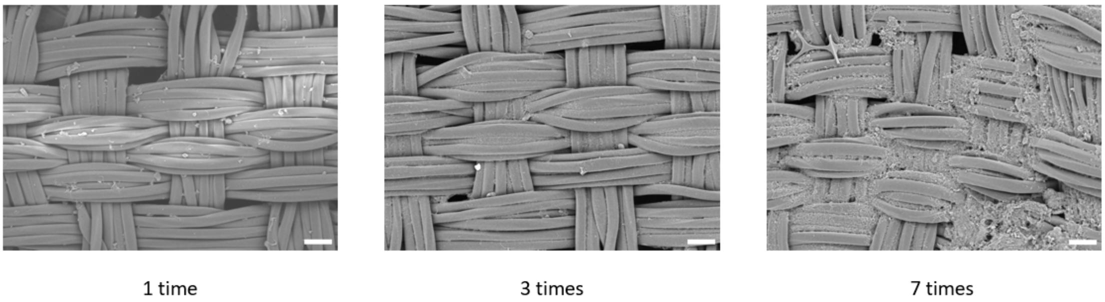

Figure S1. SEM images of the fabric with different coating times. Error bar: 50 μm.

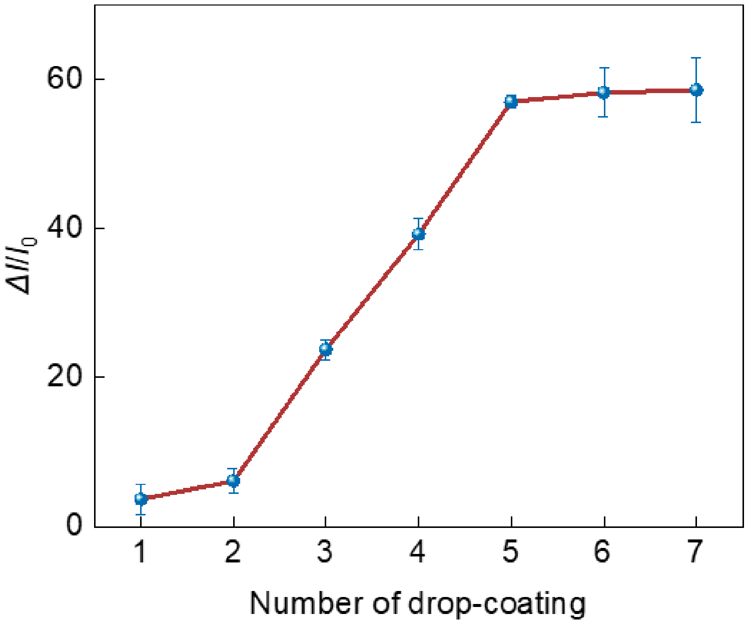

Figure S2. The current variations of the fabric-based sensor with different drop-coating times.

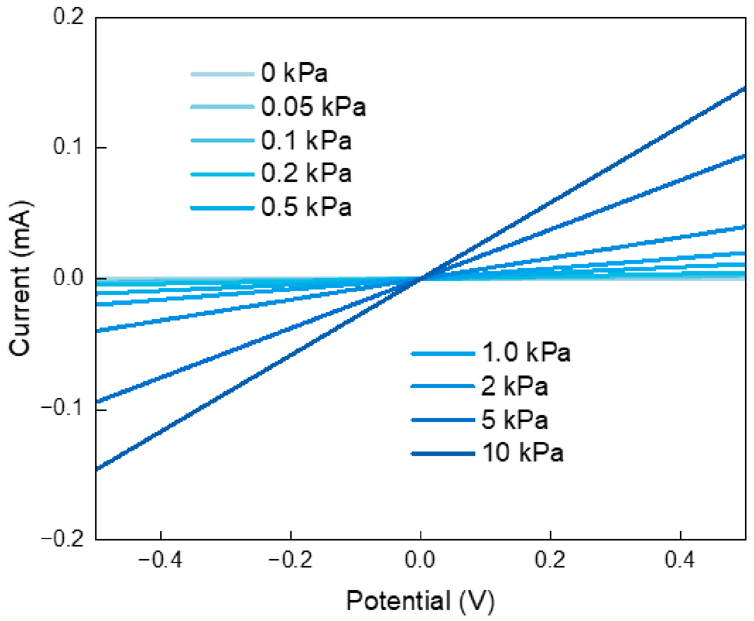

Figure S3. *I*-*V* curve under various applied pressure loads.

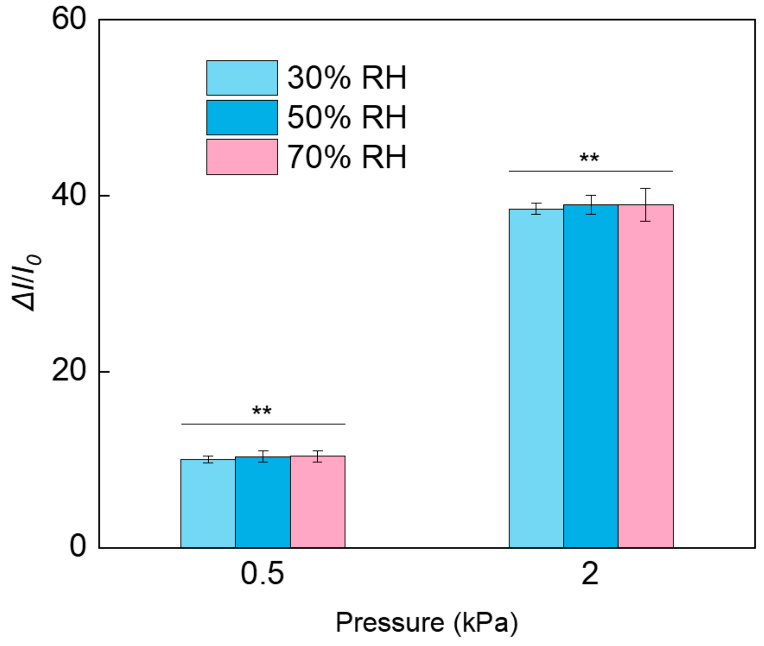

Figure S4: The signal response of the sensor in different humidity conditions.

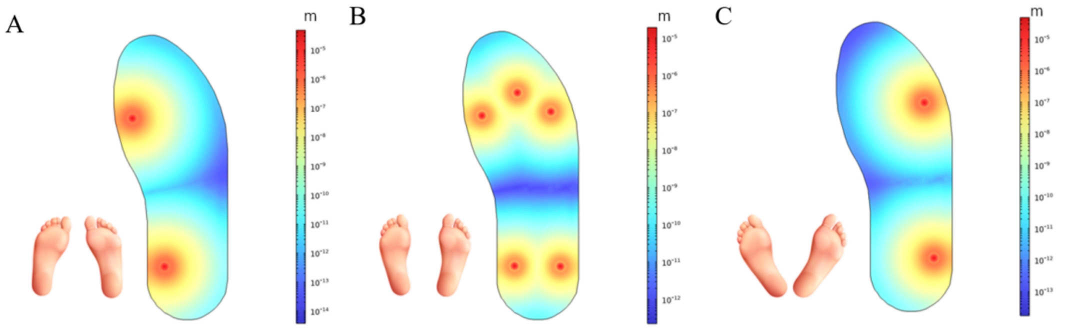

Figure S5. FEA simulation of force distribution with different walking postures. A; toe-in gait; B: normal gait; C: toe-out gait.

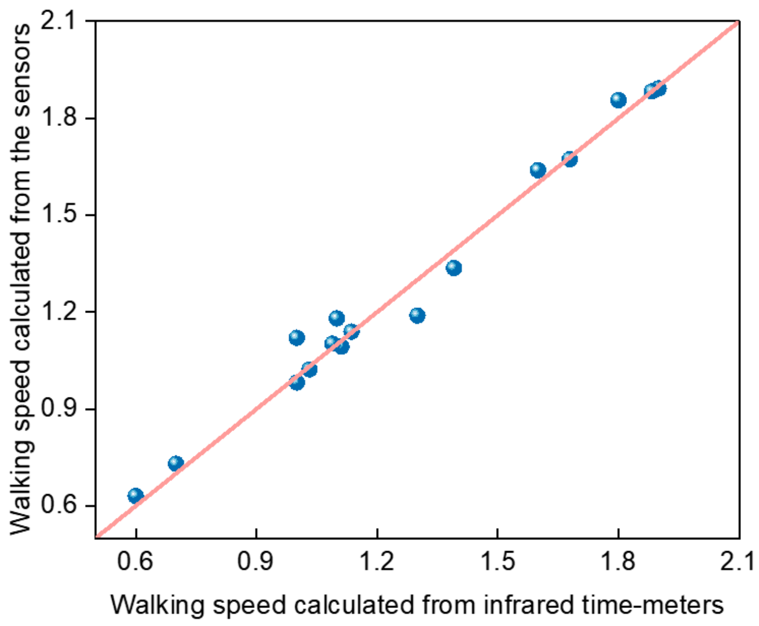

Figure S6. The correlation between walking speed calculated from the sensors and infrared time-meters.

Table 1: Performance comparison with other similar works

Detection platform	Max sensitivity	Detection range	Application	Target indicators	Flexible	Reference
Channel Crack-Designed Au@PU Sponge	0.122 kPa^{-1}	0-60 kPa	Human motion	NA	Yes	[32]
Graphene/PI nanocomposite	0.023 kPa^{-1}	0-7 kPa	NA	NA	Yes	[33]

foam						
rGO-PU sponge	0.026 kPa^{-1}	0-10 kPa	Pressure distribution	NA	Yes	[34]
CB@PU Sponge	0.036 kPa^{-1}	0-16 kPa	Human motion	NA	Yes	[35]
All textile pressure sensor	14.4 kPa^{-1}	0-20 kPa	Human motion	NA	Yes	[29]
CNT-PDMS sponge	0.033 kPa^{-1}	0-150 kPa	Human motion	NA	Yes	[36]
MXene/cotton sensor	5.3 kPa^{-1}	0-150 kPa	Human motion	NA	Yes	[37]
All paper-based sensor	39.58 kPa^{-1}	0-60 kPa	Human motion	NA	Yes	[38]
Smart EMG-based socks	NA	NA	Sarcopenia	EMG	No	[24]
Inertial sensor	NA	NA	Sarcopenia	Acceleration and angular velocity	No	[16]
Flexible-Printed Piezoelectric Sensor Array	NA	NA	Sarcopenia	Plantar Pressure	Yes	[23]
Multichannel EMG Sensor	NA	NA	Sarcopenia	EMG	Yes	[26]
Flexible wearable fabric system	18.8 kPa^{-1} (below 5 kPa)	0-30 kPa	Sarcopenia	Plantar pressure; Walking speed; Gait pattern	Yes	Our work

CB: Carbon black; PU: polyurethane; CNT: Carbon nanotube; PDMS: Polydimethylsiloxane; EMG: electromyography; NA: Not applicable

16. Kim, J.-K.; Bae, M.-N.; Lee, K.B.; Hong, S.G. Identification of Patients with Sarcopenia Using Gait Parameters Based on Inertial Sensors. *Sensors* **2021**, *21*, 1786.
23. Han, S.; Xiao, Q.; Liang, Y.; Chen, Y.; Yan, F.; Chen, H.; Yue, J.; Tian, X.; Xiong, Y. Using Flexible-Printed Piezo-electric Sensor Arrays to Measure Plantar Pressure during Walking for Sarcopenia Screening. Sensors 2024, 24, 5189.
24. Leone, A.; Rescio, G.; Giampetruzzi, L.; Siciliano, P.; editors. Smart EMG-based Socks for Leg Muscles Contrac-tion Assessment. In Proceedings of the 2019 IEEE International Symposium on Measurements & Networking (M&N), Catania, Italy, 8–10 July 2019.
26. Jin, Z.; Jian, Y.; Qin, C.; Lu, Y.; Chen, J.; Ling, X.; Wang, J.; Yang, G.; Hong, S.; Zhang, Q.; et al. Wearable Multi-channel Electromyography Sensors for Homecare Sarcopenia Diagnosis. IEEE Sens. J. 2024, 24, 33361–33370.

29. Liu, M.; Pu, X.; Jiang, C.; Liu, T.; Huang, X.; Chen, L.; Du, C.; Sun, J.; Hu, W.; Wang, Z.L. Large-Area All-Textile Pressure Sensors for Monitoring Human Motion and Physiological Signals. Adv. Mater. 2017, 29, 1703700.
32. Wu, Y.-H.; Liu, H.-Z.; Chen, S.; Dong, X.-C.; Wang, P.-P.; Liu, S.-Q.; Lin, Y.; Wei, Y.; Liu L. Channel Crack-Designed Gold@PU Sponge for Highly Elastic Piezoresistive Sensor with Excellent Detectability. ACS Appl. Mater. Interfaces 2017, 9, 20098–20105.
33. Qin, Y.; Peng, Q.; Ding, Y.; Lin, Z.; Wang, C.; Li, Y.; Xu, F.; Li, J.; Yuan, Y.; He, X.; et al. Lightweight, Superelastic, and Mechanically Flexible Graphene/Polyimide Nanocomposite Foam for Strain Sensor Application. ACS Nano 2015, 9, 8933–8941.
34. Yao, H.-B.; Ge, J.; Wang, C.-F.; Wang, X.; Hu, W.; Zheng, Z.-J.; Ni, Y.; Yu, S.-H. A Flexible and Highly Pressure-Sensitive Gra-phene–Polyurethane Sponge Based on Fractured Microstructure Design. Adv. Mater. 2013, 25, 6692–6698.
35. Wu, X.; Han, Y.; Zhang, X.; Zhou, Z.; Lu, C. Large-Area Compliant, Low-Cost, and Versatile Pressure-Sensing Platform Based on Microcrack-Designed Carbon Black@Polyurethane Sponge for Human–Machine Interfacing. Adv. Func. Mater. 2016, 26, 6246–6256.
36. Song, Y.; Chen, H.; Su, Z.; Chen, X.; Miao, L.; Zhang, J.; Cheng, X.; Zhang, H. Highly Compressible Integrated Supercapacitor–Piezoresistance-Sensor System with CNT–PDMS Sponge for Health Monitoring. Small 2017, 13, 1702091.
37. Zheng, Y.; Yin, R.; Zhao, Y.; Liu, H.; Zhang, D.; Shi, X.; Zhang, B.; Liu, C.; Shen, C. Conductive MXene/cotton fabric based pressure sensor with both high sensitivity and wide sensing range for human motion detection and E-skin. Chem. Eng. J. 2021, 420, 127720.
38. Li, A.; Xu, J.; Zhou, S.; Zhang, Z.; Cao, D.; Wang, B.; Gao, W.; Zhang, W.; Zhang, F. All-Paper-Based, Flexible, and Bio-Degradable Pressure Sensor with High Moisture Tolerance and Breathability Through Conformally Surface Coating. Adv. Funct. Mater. 2024, 2410762.
